# Intermittent Fever, Its Phenomena, Peculiarities, Types, and Treatment

**Published:** 1847-03

**Authors:** T. B. Greenley

**Affiliations:** West Point, Ky.


**Art. III.**—*Intermittent Fever, its Phenomena, Peculiarities, Types, and Treatment.* By T. B. Greenley, M.D., of West Point, Ky.

Intermittent fever was more than usually prevalent in this section of country during the past fall; in fact, comparatively few persons escaped the disease, which made its appearance about the first of August, and continued till some time in November.

The portion of country to which my practice is mostly confined is comprised principally of the bottoms of the Ohio and Salt rivers and Knob creek, together with a section of country called the *hills*, lying back a short distance from the Ohio river. This last mentioned tract of country is interspersed with a great many ponds or sinks, filled with water, which never go dry. These sinks or ponds are formed, it is supposed, by the caving in of the limestone rock which underlies this region. These ponds appear to be increasing every year, and affect the health of the country in their vicinity to a serious extent.

While there was a great increase in the number of cases of intermittent fever in this section during the past season, the disease assumed, if possible, a greater variety of forms. I have had to deal with the quotidian and double quotidian, the tertian and double tertian, the quartan and double quartan, the quintidian, weekly, malignant and simple intermittent, together with their numberless complications. The malignant form of this disease has, this season, like the simple variety, manifested itself in more than the usual proportion of cases; and likewise has presented a great variety of phenomena. Most commonly, however, it confined itself to the tertian type.

I well recollect the first case of this disease that came under my treatment. When called in, the patient was laboring under the second chill. He was vomiting incessantly, bringing up considerable quantities of bloody mucus, having something of the appearance of patches of the mucous coat of the stomach after death from inflammation and softening. He was cold up to his hips, as well as on the surface generally; suffered with great thirst, and complained of being too warm. He was perfectly in his senses, and told that he was dying. In fact, he looked very much like a dying man. There was no time to be lost. I had two boxes of mustard made into plasters, and covered him pretty well up with them, allowing them to remain on until they completely reddened the surface. This had the effect of bringing on reaction to a considerable extent, and subdued the vomiting in some degree. We now cupped him freely over the stomach, and applied a blister to the same region; the treatment entirely relieved the irritability of the stomach. In the mean time, we administered internally calomel, quinine and opium, in the form of pills, every two hours—the two latter articles in liberal quantities. This treatment proved successful, and the patient was up in a few days.

In nearly all the cases of the malignant form of intermittent fever which came under my observation the past season, great irritability of the stomach was present; and in many instances profuse quantities of blood were vomited. In fact, vomiting was the most formidable symptom we had to contend with in many of the cases. I soon found that the usual means employed to allay sickness at the stomach and vomiting were unavailing. My plan, in cases where obstinate vomiting was present, was to cup freely over the epigastrium, and if this did not succeed in arresting it, to apply a blister plaster over the same region. This treatment was adopted by myself in the first case of malignant intermittent fever that came under my observation last season, which I believe was the first that made its appearance in this region. It occurred in August. I continued to pursue this practice in all cases where the stomach was so much involved as to keep up constant efforts at vomiting. I found that it was useless to attempt to arrest this symptom by internal remedies, as nothing was retained. Occasionally it was checked temporarily by applications of mustard to the epigastrium; but to insure lasting relief, it was necessary to produce permanent counter irritation; and this was effected by cups and blisters.

This plan, with the usual internal means for preventing the recurrence of the paroxysm, was employed not only by myself, but, I believe, by the other practitioners of this place, in all cases where great irritability of the stomach was present, and with very great success; I do not remember a fatal case of the disease within the bounds of our practice.

I had two cases attended with spasms, of which the following is a short account:

*Case* 1.—Mr. G., a middle aged man, of rather delicate frame and constitution, but generally enjoying tolerably good health, was attacked violently with his second chill on the 7th of September. When I arrived, a few minutes after he was taken, he was on the bed, springing from one side to the other, with his hands applied over his abdomen. Every time the spasm came on, he gave a convulsive scream; said the cramp was in his bowels, and produced the severest pain he ever felt; great irritability of the stomach was present. Large plasters of mustard were applied over the abdomen, spine and extremities; he was cupped at the epigastrium; and internally quinine and opium in free doses, combined with a small portion of calomel, were given. He was convalescent next day, and soon got well.

*Case* 2.—Mr. C., also of middle age, of strong constitution and robust habit, was attacked about daybreak on the 28th of September with his second chill. I saw him about twelve o'clock on the same day, when reaction was thoroughly established. There was great excitement of the circulation—pulse strong, full and frequent. He was delirious, every few minutes springing from the bed, screaming with cramp in his legs, but recognising you when spoken to in an earnest manner. I bled him from the arm to the amount of thirty-two ounces before any beneficial effect was obtained; the surface then became relaxed. A good dose of opium and quinine was given, with two grains of calomel, to be repeated at intervals of two hours until three doses were taken. The severity of the spasms was greatly diminished by the loss of blood, and the delirium considerably abated.

Sept. 29. I saw the patient again this morning about ten o'clock; he was nearly well. I gave him a dose of cathartic medicine, and dismissed him.

The following is a condensed account of the case of a lady whom I was called to see on the 28th of September:

*Case* 3.—Mrs. D., aged about thirty years, was laboring under her second chill when I arrived. She vomited incessantly; complained of great pain in the uterine region, which was of a paroxysmal character, simulating exactly labor pains, but I learned on inquiry that she was not pregnant. Her hearing and vision were so impaired that she could not hear unless spoken to in a very loud voice, and though in the day, she contended that it was dark, and wished a candle lighted. The treatment was pretty much the same as in the preceding cases. I gave her opium and quinine freely, especially the former. Although she is a woman of weakly constitution and delicate health, and unused to stimulants, it required what was equivalent to six grains of opium to quiet her, and this was given her within four or five hours. Cups were applied to the epigastrium, followed by a blister.

Next morning about ten o'clock I saw her again. She was a great deal better; the vomiting had ceased; she was clear of pain, and convalescing rapidly. The next paroxysm was prevented, and she soon got well.

*Case* 4.—Mr. B., aged about thirty years, in rather delicate health, was taken with his third chill on the 10th of October. When I arrived, he was cold, and was vomiting and purging. He threw up large quantities of blood from the stomach. I cupped him freely over the stomach, and applied an extensive blister plaster to the same part. Mustard was applied to the extremities and spine; and as soon as the vomiting was checked, free doses of opium and quinine, with a small portion of calomel, were administered internally. This treatment was successful, but the patient was several days in recovering; owing to great debility, supposed to result from the loss of so much blood by vomiting. It would be proper here to remark that the usual means were employed in the above cases, as well as all that came under my treatment, to guard against the occurrence of the next paroxysm. These means were preparations of cinchona, as quinine, ext. cinchonine and quinine; with opium; together with an avoidance of exciting causes.

I found it necessary to take blood from the arm in but a single case, that of Mr. C., above mentioned. Generally speaking, the circulation was rather languid, even after reaction had ensued. In the ordinary or simple form of intermittent fever, I frequently employed venesection with great advantage. The necessity for this practice of course was indicated in such cases by the usual symptoms demanding the loss of blood, such as great power of the circulation, hot and dry skin, pain in the head, hurried respiration, etc. Where there existed much irritability of the stomach, I usually administered the medicine in the form of pill, finding it more readily retained in this form than in that of powders.

I have used what is called the impure sulphate of quinine, or precipitated extract of bark, with great success in the treatment of intermittent fever. I give about as much again by weight of this medicine as of sulphate of quinine. It has appeared to me to have a better effect when combined with diaphoretic portions of ipecacuanha or Dover's powder. I usually preceded its administration by a purgative dose of calomel and rhubarb, four or five grains of the former and fifteen of the latter, where indications for its use were present. I think this medicine has an advantage over quinine, in its being more stimulating.

Quinine scarcely ever failed to arrest the disease, but in a large number of cases it returned in from one to three weeks from the time it was arrested. Some patients were troubled with as many as six or eight relapses.

I succeeded in producing permanent cures, in some cases, with Peruvian bark and carbonate of iron, taken for some time after the paroxysms were arrested with quinine. I have also destroyed the predisposition of the system to relapse by repeating the quinine, or its equivalent, weekly, just as if the disease had been present.

There is some difficulty, however, in prevailing on some patients to adhere to a prescription of this kind, as they do not like to take medicine when they are not sick.

Arsenic could not be used with safety in many cases, owing to the presence of or tendency to irritation in the stomach and bowels.

February 10, 1847.

